# Supplementary material for: Atypical APC/C‐dependent degradation of Mcl‐1 provides an apoptotic timer during mitotic arrest
Source: EMBO J. 2018 Jul 9;37(17):e96831. doi: 10.15252/embj.201796831 (PMC6120658; doi:10.15252/embj.201796831)
Supplement: Supplementary file 1 — Expanded View Figures PDF [file EMBJ-37-e96831-s001.pdf]

Expanded View Figures

Figure EV1. Loss of FBW7 does not prevent Mcl-1 degradation during mitosis but does compromise mitotic arrest (related to Fig 1).

A Characterisation by Western blotting of DLD1 $FBW7^{+/+}$ (WT) and $FBW7^{-/-}$ (KO) cells. *denotes a non-specific band. Elevation of Myc, a substrate of Fbw7, demonstrates the effect of Fbw7 loss.

B Western blotting analysis of Mcl-1 degradation in DLD1 WT and FBW7 KO in cells arrested with 100 nM paclitaxel (upper panels). The efficacy of 100 nM paclitaxel to induction of mitotic arrest is indicated by flow cytometric analysis of histone H3 phosphorylated on Ser10 (lower panel).

C Analysis of mitotic arrest induced by treatment of DLD1 WT and FBW7 KO cells with 500 nM paclitaxel as in (B).

D YFP-Mcl-1 is degraded as endogenous Mcl-1 during a synchronised mitotic arrest.

E Live-cell imaging of the degradation of cyclin A-Venus and YFP-Nek2A during mitotic arrest induced by treatment with 830 nM nocodazole. Error bars represent ±SD, $n = 3$.

F Confirmation of siRNA knockdown of Fbw7 or APC2 and APC11 and the stabilisation of cyclin E and Myc following Fbw7 depletion in HeLa cells stably expressing YFP-Mcl-1. *denotes a non-specific band.

Source data are available online for this figure.

© 2018 The Authors

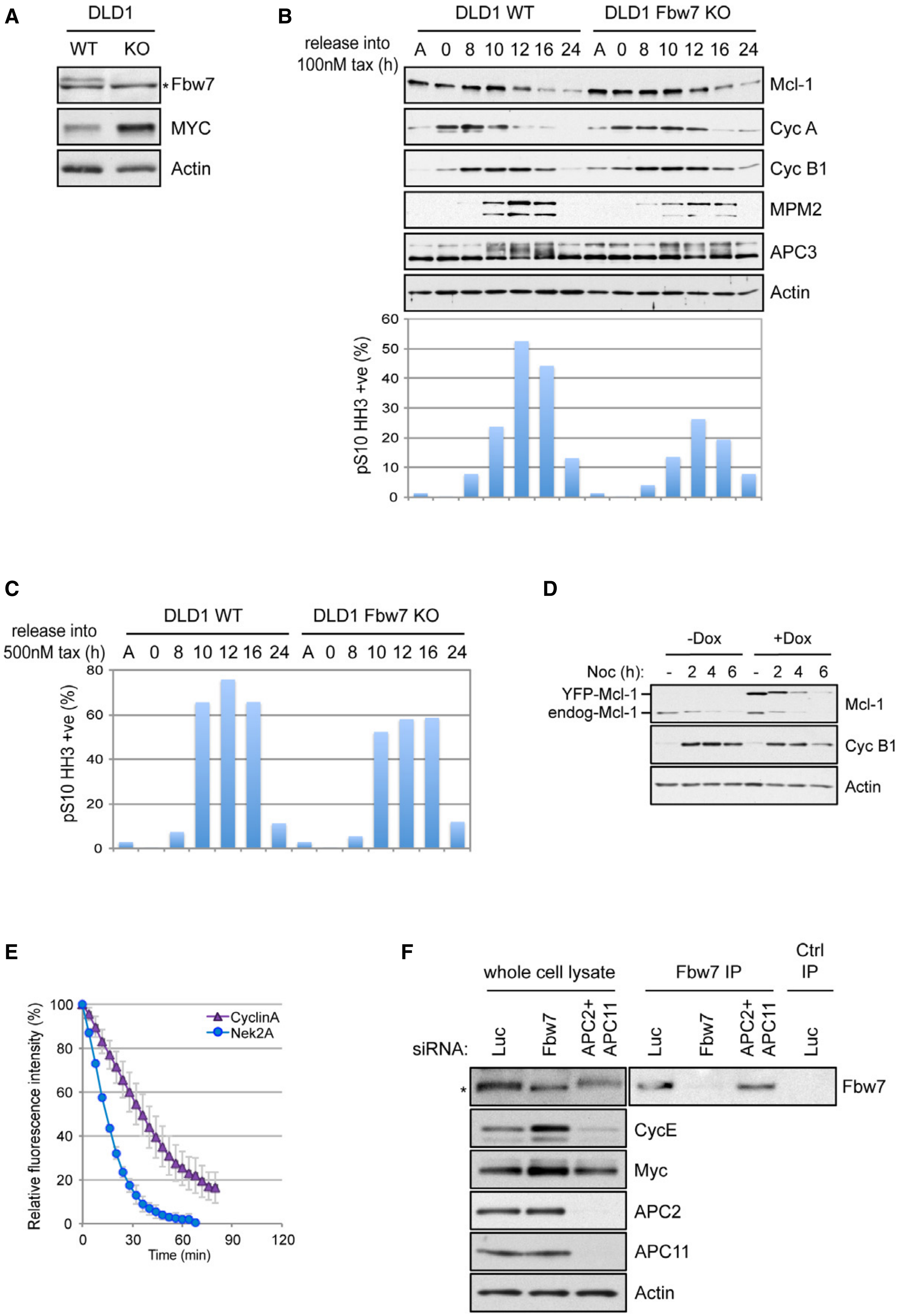
A
DLD1
WT KO
*Fbw7
MYC
Actin
B
DLD1 WT
DLD1 Fbw7 KO
release into 100nM tax (h)
A 0 8 10 12 16 24 A 0 8 10 12 16 24
Mcl-1
Cyc A
Cyc B1
MPM2
APC3
Actin
pS10 HH3 +ve (%)
C
release into 500nM tax (h)
DLD1 WT
DLD1 Fbw7 KO
A 0 8 10 12 16 24 A 0 8 10 12 16 24
pS10 HH3 +ve (%)
D
-Dox
+Dox
Noc (h): - 2 4 6 - 2 4 6
YFP-Mcl-1
endog-Mcl-1
Mcl-1
Cyc B1
Actin
E
CyclinA
Nek2A
Relative fluorescence intensity (%)
Time (min)
F
whole cell lysate
Fbw7 IP
Ctrl IP
siRNA:
Luc
Fbw7
APC2+ APC11
Luc
Fbw7
APC2+ APC11
Luc
*
Fbw7
CycE
Myc
APC2
APC11
Actin

Figure EV1.

Figure EV2. APC/C subunit requirements for Mcl-1 degradation during mitotic arrest (related to Fig 3).

A, B Confirmation of siRNA knockdown of APC3, APC8 and APC11 in HeLa cells stably expressing (A) YFP-Mcl-1 or YFP-Mcl-1ΔIR or (B) cyclin A-Venus or cyclin B1-Venus.

C, D APC3, APC8 or APC11 were knocked down by siRNA prior to treatment with nocodazole to induce mitotic arrest. The effect on the degradation of cyclin A-Venus protein in 830 nM nocodazole (C) and cyclin B1-Venus protein in 33 nM nocodazole (D) was analysed by time-lapse microscopy. The nocodazole concentration was reduced to analyse cyclin B1 degradation since cyclin B1 was stable for the duration of the experiment at a higher nocodazole concentration (830 nM, data not shown). Representative images (left) and quantification (right) are shown. Error bars represent ±SD, n = 3. Scale bar, 10 μm.

E Confirmation of siRNA knockdown of APC10 in HeLa cells stably expressing YFP-Mcl-1 WT or ΔIR, or cyclin B1-Venus.

F APC10 was knocked down by siRNA prior to treatment with nocodazole (33 nM) to induce mitotic arrest. The effect on the degradation of cyclin B1-Venus was analysed by time-lapse microscopy. Error bars represent ±SD, n = 3.

Source data are available online for this figure.

© 2018 The Authors

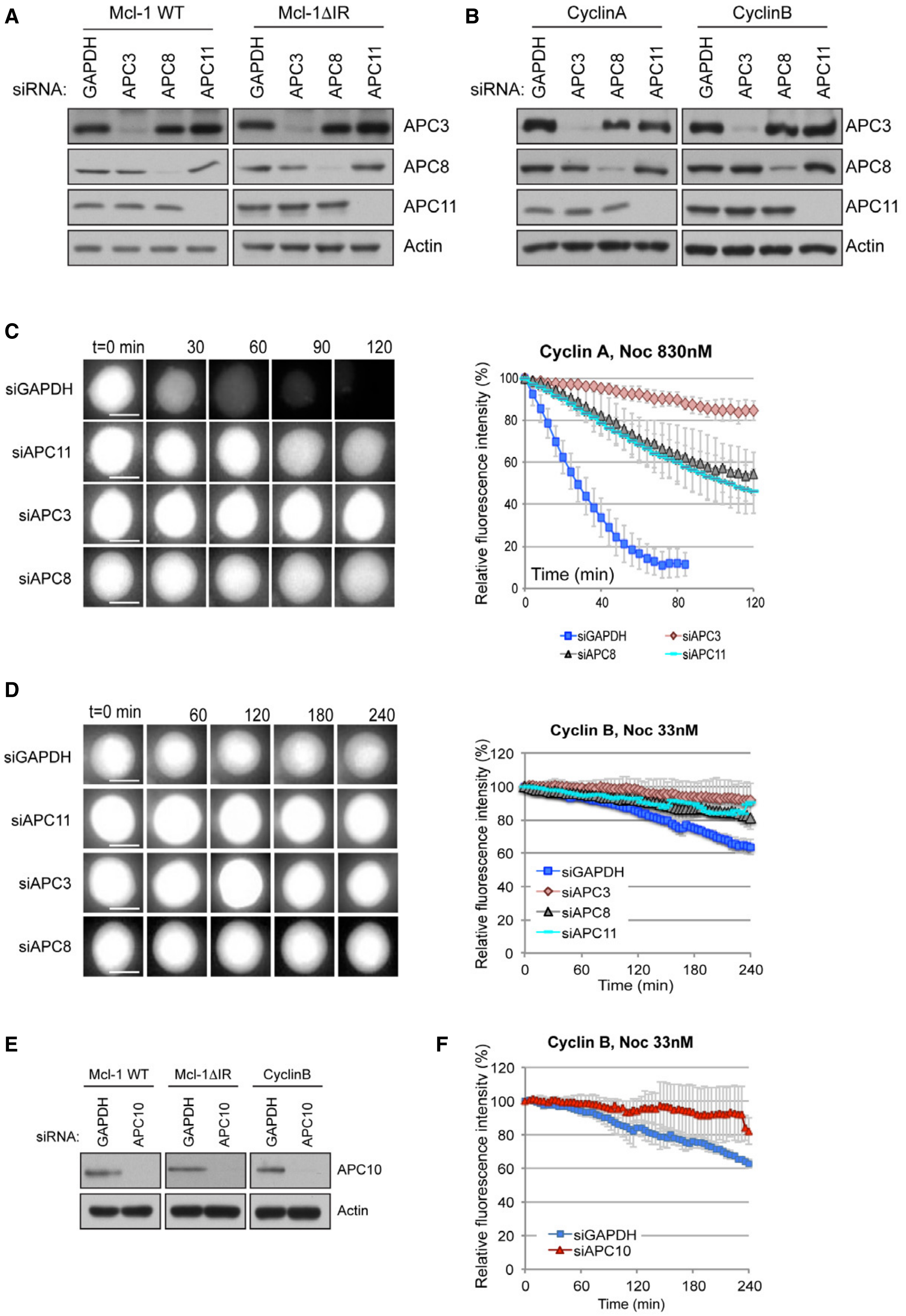
A
Mcl-1 WT
Mcl-1ΔIR
siRNA:
GAPDH
APC3
APC8
APC11
APC3
APC8
APC11
Actin
B
CyclinA
CyclinB
C
t=0 min
30
60
90
120
siGAPDH
siAPC11
siAPC3
siAPC8
Cyclin A, Noc 830nM
Relative fluorescence intensity (%)
Time (min)
D
t=0 min
60
120
180
240
Cyclin B, Noc 33nM
E
Mcl-1 WT
Mcl-1ΔIR
CyclinB
APC10
F
Cyclin B, Noc 33nM
siGAPDH
siAPC10

Figure EV2.

Figure EV3. Cdc20 and Cdh1 are not required for the degradation of Mcl-1 during mitotic arrest (related to Fig 5).

A Confirmation of siRNA knockdown of Cdc20 in HeLa cells stably expressing YFP-Mcl-1 WT.

B Western blotting of cells synchronised during mitotic arrest with nocodazole (830 nM) confirmed that the degradation of YFP-Mcl-1 protein reflected temporally that of endogenous Mcl-1 and that both were unaffected by Cdc20 depletion.

C Mcl-1 ubiquitination is inhibited by depletion of the catalytic APC/C subunits but not Cdc20. HeLa-YFP-Mcl-1 cells transfected with His-ubiquitin were depleted of APC2 and APC11 or Cdc20. Dox was added where indicated to induce expression of YFP-Mcl-1. Ubiquitinated proteins were purified and samples blotted for Mcl-1. Cell lysates (input) were also blotted with indicated antibodies to demonstrate knockdown of protein expression. Luc1 denotes luciferase siRNA transfected for 48 h as a control for siAPC2 and 11; Luc2 indicates luciferase transfection for 24 h as a control for siCdc20.

D Cdc20 and Cdh1 were knocked down singly or in combination by siRNA prior to induction of mitotic arrest with nocodazole (830 nM). The effect on the degradation of YFP-Mcl-1 was analysed by time-lapse microscopy. Error bars represent ±SD, $n = 3$. Efficiency of Cdc20 and Cdh1 knockdown is shown in the accompanying Western blot.

E Confirmation of siRNA knockdown of Cdc20 in HeLa cells stably expressing Mcl-1ΔIR or cyclin A-Venus.

F Cdc20 was knocked down by siRNA prior to treatment with nocodazole (830 nM) to induce mitotic arrest. The effect on the degradation of cyclin A-Venus was analysed by time-lapse microscopy. Error bars represent ±SD, $n = 3$. Scale bar, 10 μm.

G APC3 was knocked down by siRNA prior to treatment with nocodazole (830 nM) and reversine (500 nM) to generate a weak checkpoint. The effect on the degradation of YFP-Mcl-1ΔIR was analysed by time-lapse microscopy. Quantification of 10 individual cells from one experiment is shown. The traces are overlaid with mean values (open characters, $n = 3$). Error bars represent ±SD.

H Representative images (left panels) and quantification (right panels) of the degradation of Mcl-1ΔIR in nocodazole-treated cells depleted of Cdc20, forced to exit mitosis by inhibiting Cdk1. Cdc20 was knocked down prior to treatment of cells with nocodazole (830 nM). After 6 h, cells already arrested in mitosis were imaged by time-lapse microscopy for 20–28 min before adding Cdk1 inhibitor (R03306, 10 μM) to force mitotic exit. Cells were then imaged for another 1.5 h. Results are representative of three experiments. Scale bar, 10 μm.

I Western analysis demonstrating that Mcl-1 UTR siRNA specifically knocked down endogenous Mcl-1 and had no effect on the expression of YFP-Mcl-1 WT or ΔIR.

Source data are available online for this figure.

 © 2018 The Authors

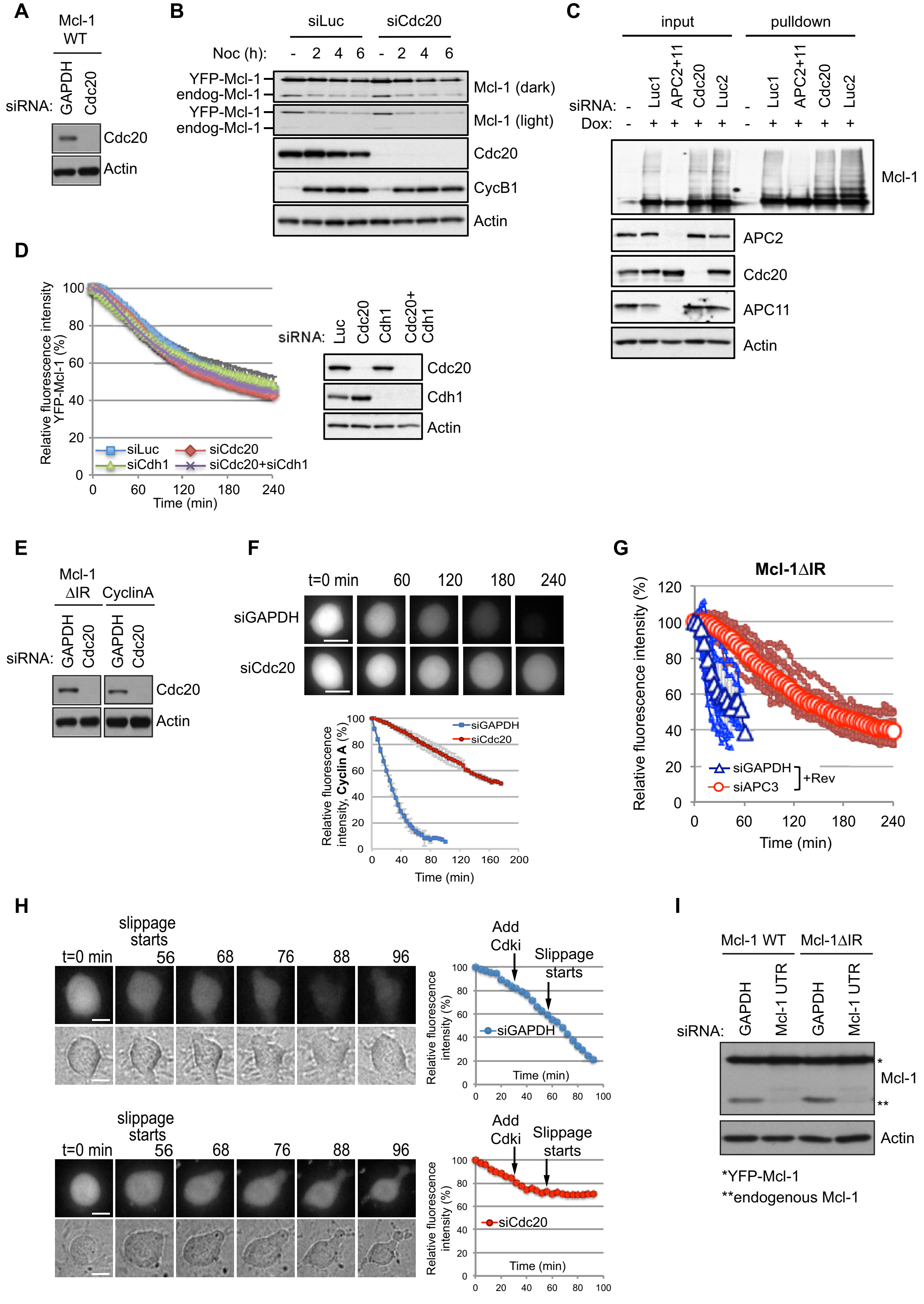
A
Mcl-1 WT
siRNA: GAPDH Cdc20
Cdc20
Actin
B
siLuc siCdc20
Noc (h): - 2 4 6 - 2 4 6
YFP-Mcl-1
endog-Mcl-1
Mcl-1 (dark)
Mcl-1 (light)
Cdc20
CycB1
Actin
C
input pulldown
siRNA: - Luc1 APC2+11 Cdc20 Luc2 - Luc1 APC2+11 Cdc20 Luc2
Dox: - + + + + - + + + +
Mcl-1
APC2
Cdc20
APC11
Actin
D
Relative fluorescence intensity YFP-Mcl-1 (%)
Time (min)
siLuc siCdc20 siCdh1 siCdc20+siCdh1
siRNA: Luc Cdc20 Cdh1 Cdc20+ Cdh1
Cdc20
Cdh1
Actin
E
Mcl-1 ΔIR CyclinA
siRNA: GAPDH Cdc20 GAPDH Cdc20
Cdc20
Actin
F
t=0 min 60 120 180 240
siGAPDH
siCdc20
Relative fluorescence intensity, Cyclin A (%)
Time (min)
G
Mcl-1ΔIR
Relative fluorescence intensity (%)
siGAPDH siAPC3 +Rev
Time (min)
H
slippage starts
t=0 min 56 68 76 88 96
Add Cdki
Slippage starts
siGAPDH
siCdc20
Relative fluorescence intensity (%)
Time (min)
I
Mcl-1 WT Mcl-1ΔIR
siRNA: GAPDH Mcl-1 UTR GAPDH Mcl-1 UTR
Mcl-1
Actin
*YFP-Mcl-1
**endogenous Mcl-1

Figure EV3.

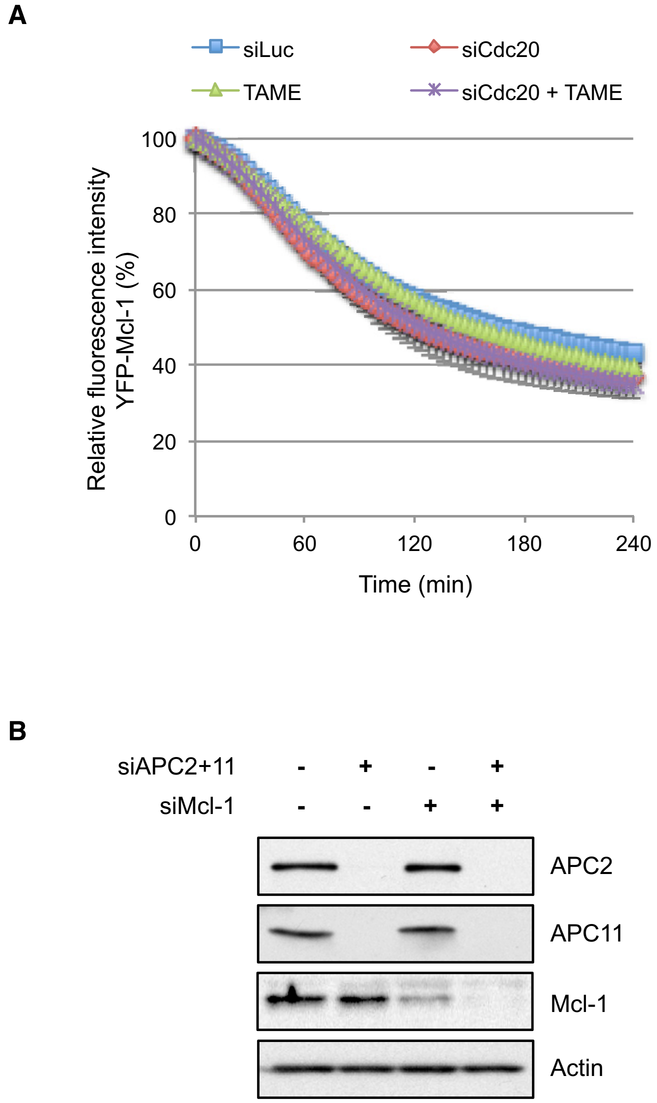

Figure EV4. Knockdown of Cdc20 and APC/C subunits (related to Fig 6).

A Cdc20 was knocked down 24 h prior to the addition of proTAME (10 μM) where indicated. During the subsequent mitotic arrest, the effect on the degradation of YFP-Mcl-1 was analysed by time-lapse microscopy. Error bars represent ±SD, n = 3.

B Western blot analysis demonstrating the effective knockdown of APC2, APC11 and Mcl-1 in RPE-1 cells.

Source data are available online for this figure.

 © 2018 The Authors
