# Supplementary figures and images for: Atypical APC/C‐dependent degradation of Mcl‐1 provides an apoptotic timer during mitotic arrest
Source: EMBO J. 2018 Jul 9;37(17):e96831. doi: 10.15252/embj.201796831 (PMC6120658; doi:10.15252/embj.201796831)

Figure EV1

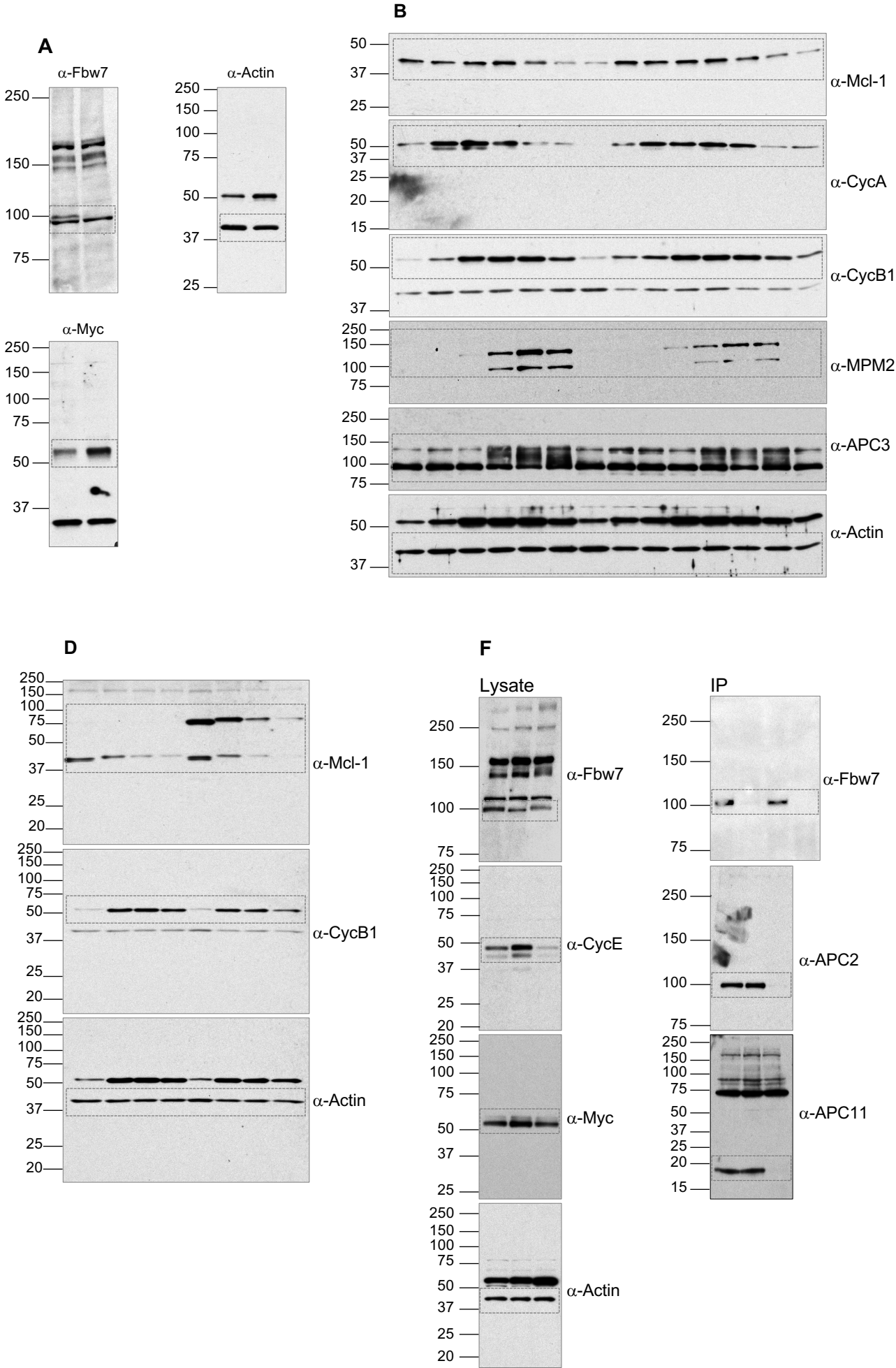

Supplement: Supplementary file 2 — Source Data for Expanded View [file EMBJ-37-e96831-s004.zip › Source_data_3-6-18_Fig_EV1.pdf]

Figure EV2

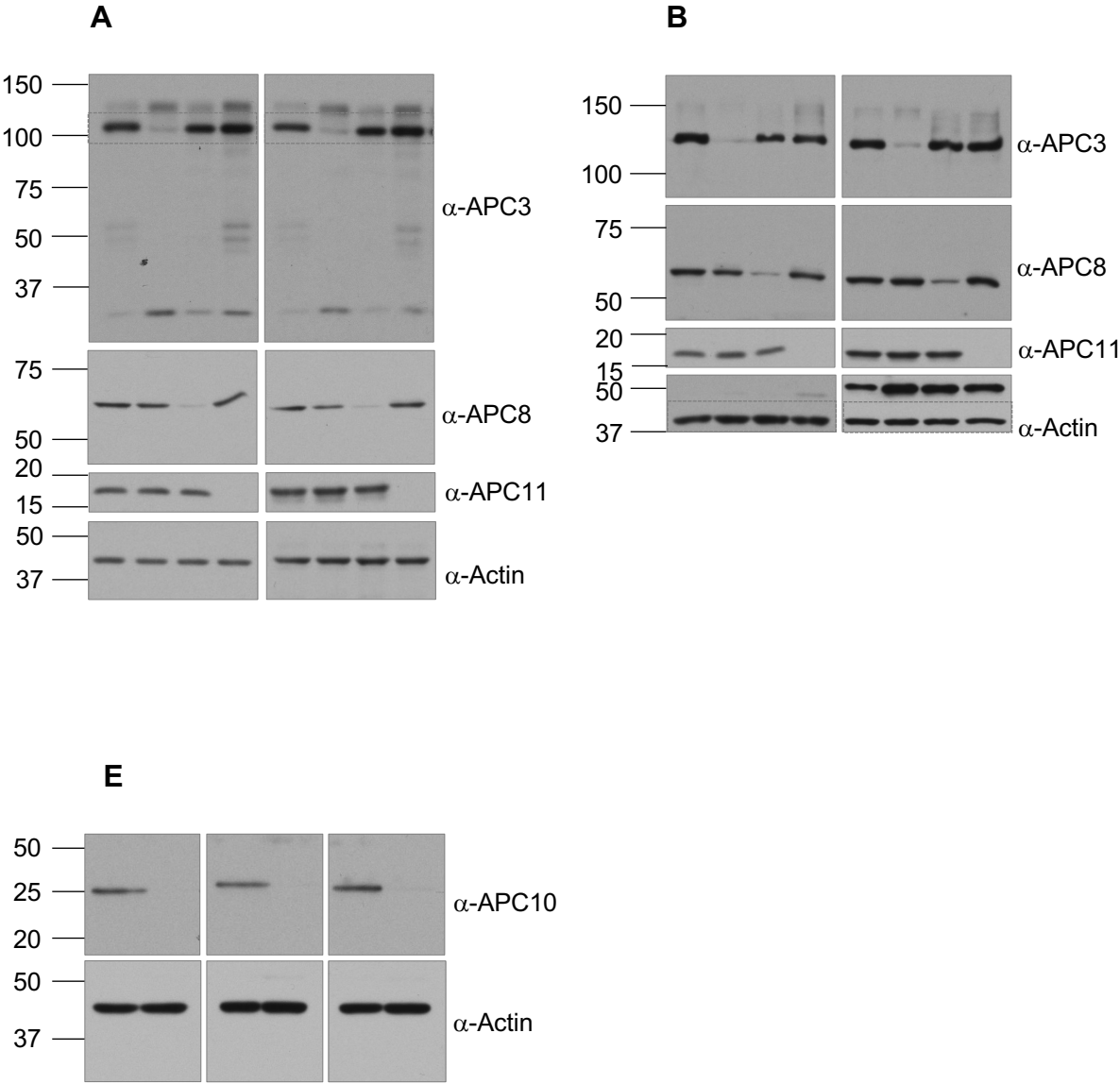

Supplement: Supplementary file 2 — Source Data for Expanded View [file EMBJ-37-e96831-s004.zip › Source_data_3-6-18_Fig_EV2.pdf]

Figure EV3

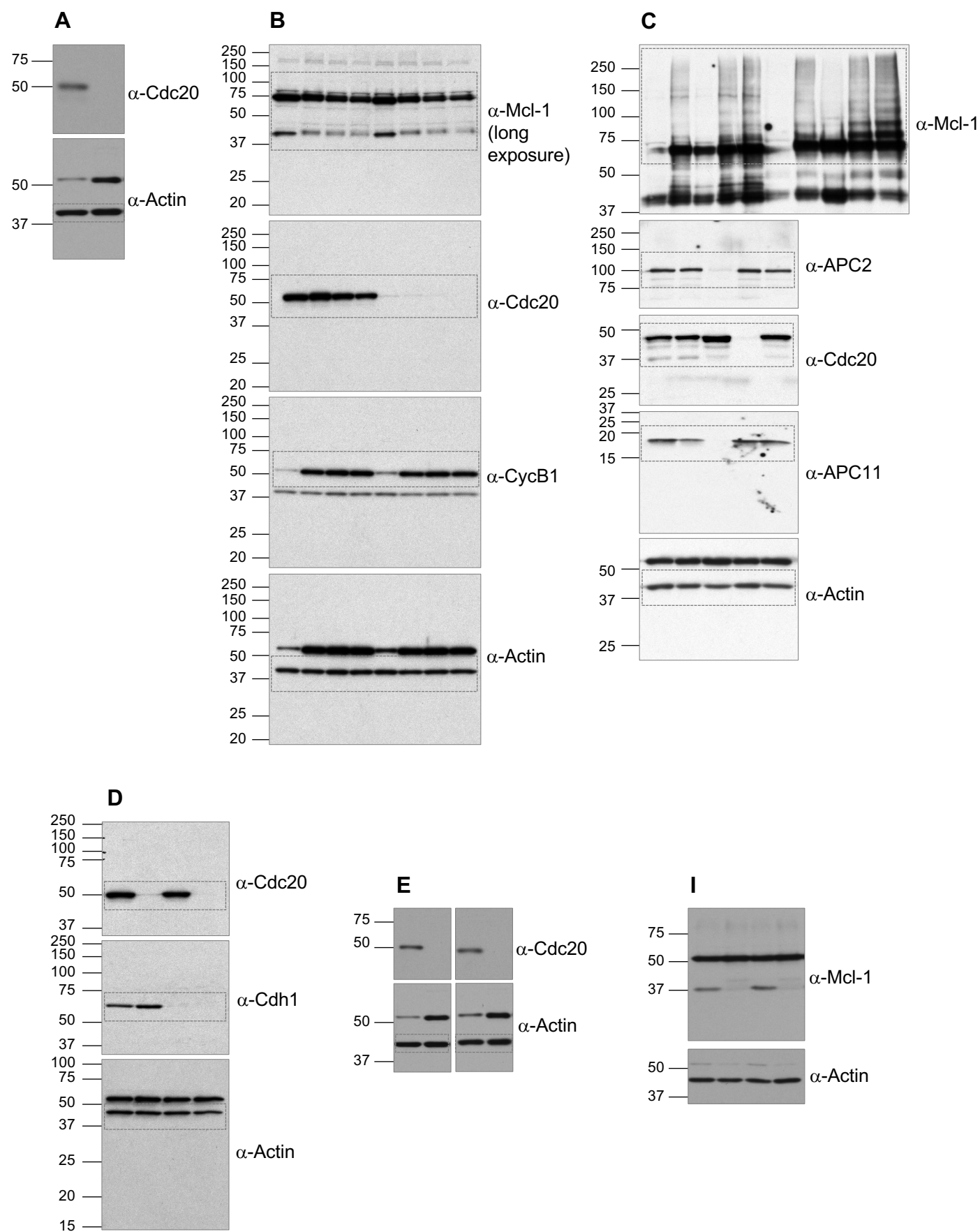

Supplement: Supplementary file 2 — Source Data for Expanded View [file EMBJ-37-e96831-s004.zip › Source_data_3-6-18_Fig_EV3.pdf]

Figure EV4

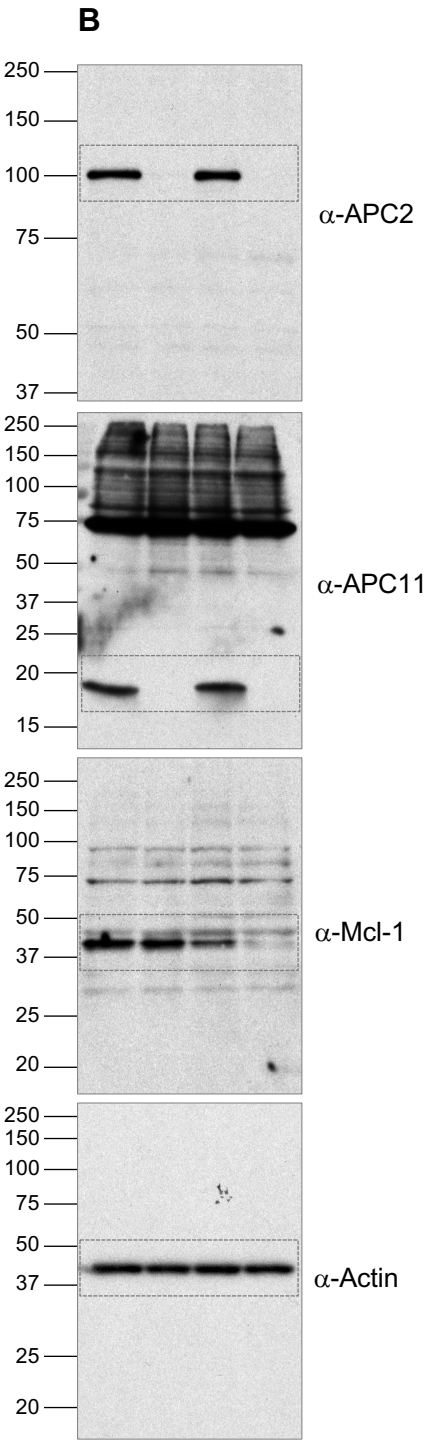

Supplement: Supplementary file 2 — Source Data for Expanded View [file EMBJ-37-e96831-s004.zip › Source_data_3-6-18_Fig_EV4.pdf]

Figure 1

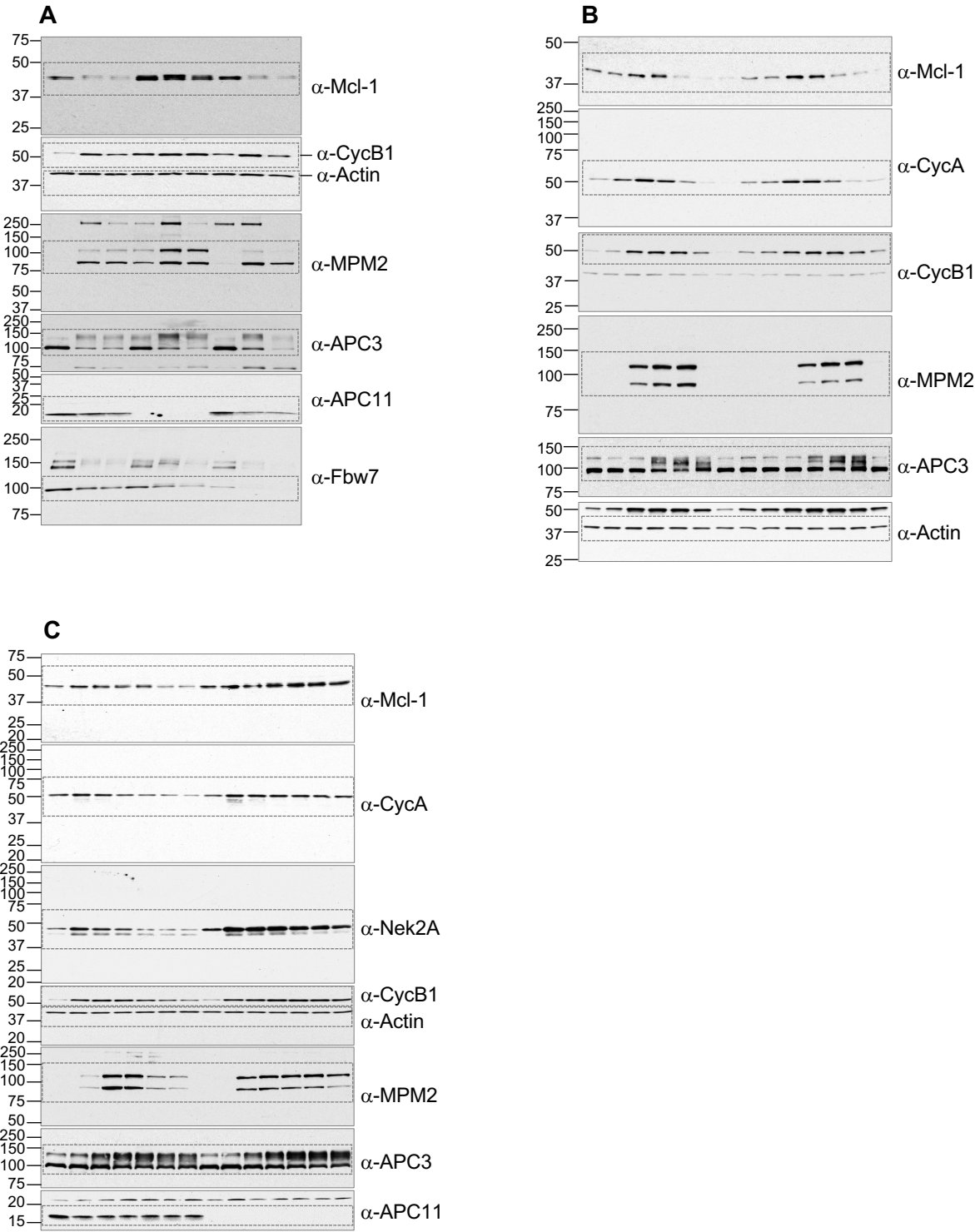

Supplement: Supplementary file 4 — Source Data for Figure 1 [file EMBJ-37-e96831-s002.pdf]
