# Supplementary material for: Atypical APC/C‐dependent degradation of Mcl‐1 provides an apoptotic timer during mitotic arrest
Source: EMBO J. 2018 Jul 9;37(17):e96831. doi: 10.15252/embj.201796831 (PMC6120658; doi:10.15252/embj.201796831)
Supplement: Supplementary file 5 — Source Data for Figure 2 [file EMBJ-37-e96831-s003.pdf]

Figure 2

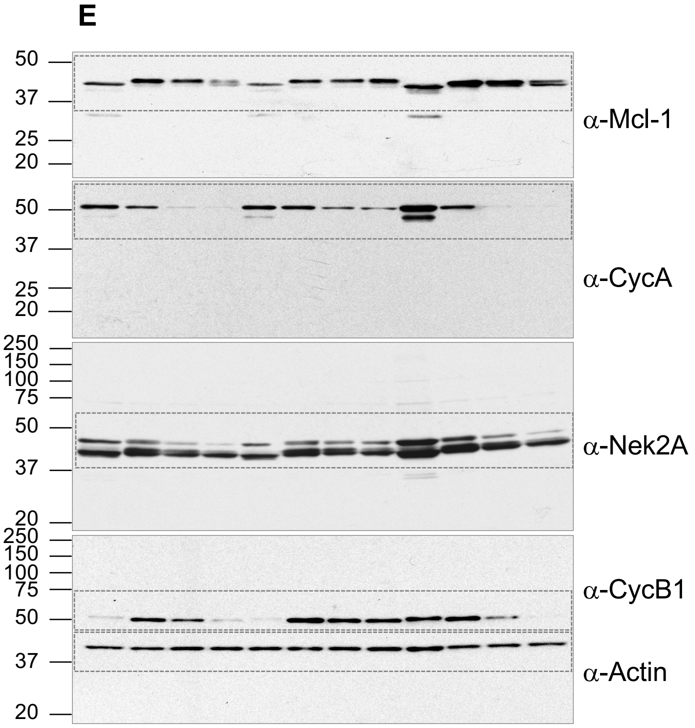
E
50
37
25
20
α-Mcl-1
50
37
25
20
α-CycA
250
150
100
75
50
37
20
α-Nek2A
250
150
100
75
50
37
20
α-CycB1
α-Actin
